# SG-APSIC1166: Strategies to reduce hospital-onset *Clostridioides difficile* infections in an acute-care hospital in Singapore

**DOI:** 10.1017/ash.2023.87

**Published:** 2023-03-16

**Authors:** Shi Yun Foo, Li JIe, Foo Shi Yun, Chai Hairu, Theresa Cabahug, An Rong Yan, Wu Tuodi, Suhailah Binte Nasir, Harminder Kaur

**Affiliations:** Changi General Hospital, Singapore; Changi General Hospital, Singapore; Changi General Hospital, Singapore; Changi General Hospital, Singapore; Changi General Hospital, Singapore; Changi General Hospital, Singapore; Changi General Hospital, Singapore; Changi General Hospital, Singapore; Changi General Hospital, Singapore

## Abstract

**Objectives:** Control of *Clostridioides difficile* infections (CDIs) in healthcare facilities presents significant challenges to infectious disease physicians, infection prevention and control practitioners, and environmental services staff. CDI is a common cause of infectious diarrhea and is associated with significant morbidity, mortality, and healthcare cost. A high infection rate was documented in our institution in 2017, higher than the national infection rate. Strategies to reduce hospital-onset CDI were implemented after review of international guidelines and relevant literature. The impact on hospital-onset CDI was assessed. **Methods:** The following strategies were implemented beginning early in 2018: (1) contact precautions for patients with diarrhea; (2) early recognition and diagnosis of *C. difficile* infection; (3) prompt isolation of *C. difficile* patients; (4) emphasis on hand hygiene and contact precautions; (5) enhanced environmental cleaning with chlorine-based disinfectant and use of UV-C and ionized hydrogen peroxide for equipment disinfection; (6) enhanced cleaning and disinfection using sporicidal wipes for shared high-risk equipment; (7) audit and feedback regarding compliance with practices and environmental cleaning; and (8) collaboration with antibiotics stewardship program (ASP) to reduce inappropriate antibiotic use. Hospital-onset CDI cases were tracked by infection prevention and control nurses using definitions from the Singapore Ministry of Health. **Results:** In total, 135 hospital-onset *C. difficile* infection cases occurred in 2017, a rate of 4.2 per 10,000 patient days. This rate gradually decreased to 3.0 in 2018 and to 2.3 in 2020, with an average of 87 infections per year. This rate further decreased to 1.8 infections per 10,000 patient days in 2021, with 61 clinical infections. **Conclusions:** Using multimodal strategies, CGH achieved a gradual and steady reduction in hospital-onset CDI over several years. These strategies require close collaboration among various departments to achieve the desired outcome.

